# Ellagic Acid Derivatives from *Terminalia chebula* Retz. Downregulate the Expression of Quorum Sensing Genes to Attenuate *Pseudomonas aeruginosa* PAO1 Virulence

**DOI:** 10.1371/journal.pone.0053441

**Published:** 2013-01-08

**Authors:** Sajal Sarabhai, Prince Sharma, Neena Capalash

**Affiliations:** 1 Department of Microbiology, Panjab University, Chandigarh, India; 2 Department of Biotechnology, Panjab University, Chandigarh, India; Laurentian University, Canada

## Abstract

**Background:**

Burgeoning antibiotic resistance in *Pseudomonas aeruginosa* has necessitated the development of anti pathogenic agents that can quench acylhomoserine lactone (AHL) mediated QS with least risk of resistance. This study explores the anti quorum sensing potential of *T. chebula* Retz. and identification of probable compounds(s) showing anti QS activity and the mechanism of attenuation of *P. aeruginosa* PAO1 virulence factors.

**Methods and Results:**

Methanol extract of *T. chebula* Retz. fruit showed anti QS activity using *Agrobacterium tumefaciens* A136. Bioactive fraction (F7), obtained by fractionation of methanol extract using Sephadex LH20, showed significant reduction (p<0.001) in QS regulated production of extracellular virulence factors in *P. aeruginosa* PAO1. Biofilm formation and alginate were significantly (p<0.05) reduced with enhanced (20%) susceptibility to tobramycin. Real Time PCR of F7 treated *P. aeruginosa* showed down regulation of autoinducer synthase (*lasI* and *rhlI*) and their cognate receptor (*lasR* and *rhlR*) genes by 89, 90, 90 and 93%, respectively. Electrospray Ionization Mass Spectrometry also showed 90 and 64% reduction in the production of 3-oxo-C_12_HSL and C_4_HSL after treatment. Decrease in AHLs as one of the mechanisms of quorum quenching by F7 was supported by the reversal of inhibited swarming motility in F7-treated *P. aeruginosa* PAO1 on addition of C_4_HSL. F7 also showed antagonistic activity against 3-oxo-C_12_HSL-dependent QS in *E. coli* bioreporter. *C. elegans* fed on F7-treated *P. aeruginosa* showed enhanced survival with LT50 increasing from 24 to 72 h. LC-ESI-MS of F7 revealed the presence of ellagic acid derivatives responsible for anti QS activity in *T. chebula* extract.

**Conclusions:**

This is the first report on anti QS activity of *T. chebula* fruit linked to EADs which down regulate the expression of *lasIR* and *rhlIR* genes with concomitant decrease in AHLs in *P. aeruginosa* PAO1 causing attenuation of its virulence factors and enhanced sensitivity of its biofilm towards tobramycin.

## Introduction

*P. aeruginosa* is the major cause of secondary infections in immunocompromised patients with cystic fibrosis, burn wound and HIV causing maximum morbidity and mortality [1]. It is a clinically important opportunistic pathogen responsible for 57% of total nosocomial infections [2]. To facilitate the establishment of infection, *P. aeruginosa* produces both cell-associated and extracellular virulence factors globally regulated by well defined quorum sensing systems arranged in hierarchical manner with *las* system at the top, positively controlling the activity of *rhl* system [3]. The *las* system utilizes N-(3-oxododecanoyl)-L-homoserine lactone (3-oxo-C_12_HSL) whereas *rhl* system functions by means of N-butanoyl-L-homoserine lactone (C_4_HSL) as the signal molecules [4]. Intermediate between the two is the quinolone system which utilizes 2-heptyl-3-hydroxy-4-quinolone as the signal molecule [5]. Pyocyanin and rhamnolipids production is controlled by *rhlIR* system whereas elastase and proteolytic activities by *lasIR* system [6]. *P. aeruginosa* also adopts biofilm mode of growth that is regulated jointly by *lasIR* and *rhlIR* system [7] making it recalcitrant to various antimicrobial treatments.

Antibiotic treatment for *P. aeruginosa* infection includes the administration of either single β-lactam antibiotic or combination therapy including tobramycin or colistin with ceftazidime [8]. However, high level of allergy to β-lactams and emergence of resistant bacteria limits their use for *P. aeruginosa* infections [9]. This necessitates focusing on alternative strategies like using QS inhibitory agents that may improve the management of *P. aeruginosa* infection more effectively. Plant derived compounds have been used to treat microbial infections for centuries and are supposed to be safe for human consumption [10]. Screening of plant-derived compounds may facilitate the discovery of compounds that attenuate bacterial pathogenesis by interfering with QS systems and render pathogenic bacteria non-virulent without affecting their viability. This will generate less pressure for the evolution of resistance as compared to antibiotic therapy. Halogenated furanone compounds from marine alga *Delisea pulchra* reduces the cellular concentration of transcriptional regulator *luxR* of *Vibrio fischeri* cloned in *E. coli* [11]. Curcumin, from *Curcuma longa* [12], Ajoene from *Allium sativum* [13], Iberin from *Armoracia rusticana* [14] attenuate *P. aeruginosa* virulence by downregulating the expression of QS genes. Bioreporter strains with promoters of QS genes fused with *lacZ* showed downregulation of QS genes on treatment with aqueous extracts of *Conocarpus erectus*, *Bucida buceras* and *Callistemon viminalis* [15]. Plant polyphenols like epigallocatechin, ellagic acid, tannic acid [16] and chemically synthesized 4-Nitro-pyridine–N-oxide [17], S-adenosylhomocysteine [18], isothiocyanate [19] were capable of antagonizing AHL dependent QS in bioreporter strains. However, their toxicity in mammalian cells limits their use as drugs [20].

*T. chebula* Retz. belongs to Combretaceae family [21] and is commonly known as harad or black myroblans. A very well known ayurvedic formulation in India known as “Triphala” contains equal parts of *T. chebula*, *T. bellerica* and *Embilica officinalis* and has been scientifically proven to promote immunity, health and longevity [22]. Organic and aqueous extracts of *T. chebula* exhibit antioxidant [23], antimicrobial [24], antianaphylactic [25], antidiabetic [26], antimutagenic [27], anticancerous [28], apoptotic [29], anticaries [30], antifungal [31] and antiviral [32] activities. *T. chebula* fruit extract is effective antimicrobial against methicillin resistant *Staphylococcus aureus* and trimethoprim-sulphamethoxazole resistant uropathogenic *E. coli* strain [33]. To the best of our knowledge, *Terminalia* species have not been explored for anti QS activity. However, tannin rich fraction of *T. cattappa* has been shown to inhibit QS regulated violacein production in *Chromobacterium violaceum* JCM1249 and QS controlled biofilm maturation and LasA staphylolytic activity in *P. aeruginosa* ATCC 10145 [34]. The broad spectrum of activities in *T. chebula* is attributed to the presence of different types of phytochemicals where hydrolysable tannins contributed 40% of the total content that includes simple gallate esters, ellagic acid derivatives and glycosides, and various ellagitannins [35]. This study explores the anti QS potential of *T. chebula* fruit for attenuation of virulence factors of *P. aeruginosa* PAO1 and identification of compounds (s) responsible for the activity. Mechanism of anti QS activity has also been elucidated.

## Materials and Methods

Bacterial strains, plasmids and culture conditions are described in Table 1.

**Table 1:** Bacterial strains and plasmids used in this study.

Strain	Genotype or phenotype	Growth conditions	Reference or source
***P. aeruginosa***			
**PAO1**	Wild type	LB or bactopeptone at 37°C	36
**PAOJP2**	*lasI^−^rhlI^−^* derivative of PAO1*;*Tet^r^Hg^r^	LB or bactopeptone at 37°C;Tetracycline 50 µg/ml,HgCl_2_ 15 µg/ml	37
**GFP tagged PAO1**	*pSM2472* with *gfp*	LB at 37°C	38
***A.tumefaciens*** ** A136**	*pCF372* with *traI::lacZ* and *pCF218*with *traR*;Spect^r^,Tet^r^	LB at 30°C;Spectinomycin 50 µg/ml,Tetracycline 5 µg/ml	39
***C. violaceum*** ** O26**	Mini Tn5 mutant of CV31532;Km^r^	LB at 30°C;Kanamycin 30 µg/ml	40
***E.coli***			
**OP50**	Wild type food for *C. elegans*	LB or Nematode Growth Medium at 37°C	CGC centre,University of Minnesota,Twin cities, USA
**DH5α (** ***pSC1*** **1+ ** ***pJN105L*** **)**	P*lasI::lacZ*; *lasR* in pJN105;Gm^r^ amp^r^	LB at 37°C;Ampicllin 100 µg/ml,Gentamycin 15 µg/ml	41, 42

### Preparation of Plant Extract

Dried fruit of *T. chebula,* confirmed as *T. chebula* Retz. by National Institute of Scientific Communication And Information Resource (NISCAIR), New Delhi, India, was ground to fine powder and extracted with water and methanol in Soxhlet apparatus for 10–12 h, separately. The extracts were air dried and reconstituted in water and methanol, respectively.

### Disc Diffusion Assay for Anti QS Activity

A136 was used as biosensor strain for disc diffusion assay [43]. 10 µl of aqueous and methanol extracts of *T. chebula* were used in the assay with curcumin (3 µg/ml) [12], methanol and water as controls.

The integrity of AHLs in the presence of *T. chebula* extract was also checked using biosensor A136. AHLs were extracted from 100 ml of cell free supernatant of *P. aeruginosa* PAO1 using acidified ethyl acetate [44] and dissolved in 100 µl of DMSO. 20 µl of AHLs were incubated with 0.5 mg/ml of *T. chebula* extract for overnight at 37°C. AHLs were re-extracted after incubation and AHL mediated β-galactosidase activity in biosensor A136 was estimated [45].

### Separation of Bioactive Fraction from the Methanol Extract of *T. chebula*

4.0 mg of methanol extract, dissolved in ethanol, was loaded onto the Sephadex LH 20 column (GE healthcare) (30×4 cm with i.d. 2 cm) and fractionation was conducted by successive elution of sample with increasing concentration of methanol (0, 5, 10, 20, 30, 50 and 100%) in ethanol (100 ml of each solvent). Individual fractions (F1–F7) were dried at 30°C and re-suspended in 1 ml of methanol. Phytochemical analysis of the fractions was done for the presence of terpenoides, flavonoids, saponins, tannins and alkaloids [46]. The fraction that inhibited the production of violacein pigment in biosensor CVO26 [47] was used for further work.

### Quantitation of Extracellular Virulence Factors of *P. aeruginosa* PAO1

Overnight grown culture of *P. aeruginosa* PAO1 was diluted with fresh 2% bactopeptone (1∶100) and incubated at 37°C for 16 h at 150 rpm. PAOJP2, autoinducer mutant (*lasI^−^rhlI^−^*) was taken as negative control. Cell free supernatant was used for quantification of virulence factors. Pyocyanin pigment was extracted from culture supernatant (5 ml) using chloroform in the ratio of 3∶2 and re-extracted with 1.0 ml of 0.2 M HCl and absorbance was read at 540 nm [1]. 250 µl elastin congo red solution (5 mg/ml in 0.1 M Tris-HCl pH 8;1 mM CaCl_2_) was incubated with 750 µl cell free supernatant at 37°C for 16 h at 200 rpm. The mixture was centrifuged at 3000 g for 10 min and absorbance was read at 490 nm to estimate elastase activity [47]. Rhamnolipids were quantitated by adjusting the pH of the culture supernatant to 2 with HCl and the resultant suspension was centrifuged at 8000 g for 10 min. Absorbance was read at 570 nm [48]. Protease activity was determined using 2% azocasein solution prepared in 50 mM phosphate buffer saline (PBS), pH 7. The substrate and culture supernatant were incubated at 37°C in 1∶1 ratio for 1 h in a reaction volume of 400 µl. The reaction was stopped by the addition of 500 µl of 10% trichloroacetic acid and centrifuged at 8000 g for 5 min to remove residual azocasein. The absorbance of supernatant was read at 400 nm [15]. All absorbance values were reported as OD of virulence factors per growth OD_600 nm_ to normalize the effect of bioactive fraction on bacterial growth.

### Alginate Production

*P. aeruginosa* PAO1 was grown in 10 ml LB for 3 days at 37°C in 35 mm petri plate under static conditions to form biofilm. The exhausted medium in the plate were collected and loosely adhered bacterial cells were removed by repeated washing with 0.85% saline. Alginate in culture supernatant was precipitated by 2% cetylpyridinium chloride and quantified by carbazole reagent [49].

### Biofilm Formation

Biofilms were developed in 96 well polystyrene microtiter plate. 200 µl of *P. aeruginosa* (OD600 nm ∼1) culture in LB broth with 1% glycerol was incubated for 24 h at 37°C under static conditions. The supernatant surrounding the biofilm was collected and planktonic cells were quantitated by serial dilution method. Thereafter, biofilm was given three washings with PBS (50 mM, pH 7) to wash off loosely adhered planktonic bacterial cells. Subsequently, the biofilm was fixed with 200 µl of methanol for 15 min, air dried and stained with 200 µl of 0.5% (w/v) crystal violet for 15 min. The plate was washed with PBS (50 mM, pH 7) three times to remove excessive stain. 200 µl of 95% (v/v) ethanol was added to extract bound crystal violet and Biofilm Index was tabulated as OD _570 nm/600 nm_ [50].

For visualizing biofilm, sterilized glass cover slips were immersed in LB broth containing 1% glycerol, inoculated with 1% of overnight grown GFP tagged *P. aeruginosa* [38] in a 35 mm petri plate and incubated at 37°C under static conditions. The medium was changed after every 24 h for 3 days. The coverslips were washed with PBS (50 mM, pH 7) and stained with 20 µM Propidium Iodide. Confocal Laser Scanning Microscope (CLSM) images of biofilm were observed under 63X magnification and analyzed with Neiss viewer image analysis software.

### *C. elegans* Killing Assay

The wild type *C. elegans* (Bristol) N2 hermaphrodite strain was used as *in vivo* model system. Worms were synchronized by hypochlorite treatment of gravid adults. Synchronized worms were grown to L4 or young adult stage by incubating them at 25°C in Nematode Growth Medium (NGM) for killing assays. BHI agar plates were seeded with 10 µl of overnight culture of *E. coli* OP50 or *P. aeruginosa* PAO1 or PAOJP2 (*lasI* ^−^ *rhlI* ^−^) and incubated at 37°C for 24 h to form lawn of bacteria [51]. Nematodes were washed off from stock plates and suspended in a minimal volume of M9 buffer (pH 6.5). 20 adult nematodes were picked and placed onto the bacterial lawn, incubated at 25°C and were observed for killing after every 24 h for 7 days. The number of worms that survived was tabulated to observe change in LT_50_ value (time required to kill 50% of worms). Experiments were performed in triplicates. Killing curves represent the mean of three separate experiments. Bacterial population inside nematode gut was determined by the method described by Rudrappa and Bias [12].

### Mechanism of Anti QS Activity of Bioactive Fraction

#### Expression of QS genes

*P. aeruginosa* PAO1 was treated with bioactive fraction and total RNA was extracted by TRIZOL reagent (Sigma). First strand cDNA synthesis was done as per the manufacturers protocol (Fermentas). qRT PCR was done using SYBR green mastermix (Fermentas). In 10 µl reaction mixture, 5 µl of SYBR green mastermix, 100 ng of cDNA, 5 µM target gene primers (*lasI*, *lasR*, *rhlI* or *rhlR*) and 1 µM 16s rRNA primers (internal housekeeping gene) were used [52]. The qRT PCR was done using Eppendorf real plex system with two step PCR programme: 95°C for 10 min, (denaturation at 95°C for 15s and annealing at 60°C for 1 min) X40 cycles. Relative expression of gene (RQ) was calculated by 2^−ΔΔct^ and percent reduction was calculated as (1-RQ) X 100.

#### AHLs production

AHLs were extracted from 20 ml of cell free supernatant *P. aeruginosa* PAO1 treated with bioactive fraction [44] and subjected to Electrospray Ionization Mass Spectrometry (ESI-MS) to determine 3-oxo-C_12_HSL and C_4_HSL content. Sample was directly injected into a Finnegan Navigator with the nebulizer tip at 250°C and 4.52 kV. The cone voltage was 5 kV. The scans were averaged over 0.5–1.0 min (15–30 scans). Mass spectrum was observed for various m/z peaks of AHLs [53] and for change in their relative peak intensity.

#### Reversal of swarming motility

2 µl overnight grown culture of *P. aeruginosa* (OD_600 nm_ ∼1) was inoculated on swarming plates (0.5% LB agar) containing bioactive fraction alone and in combination with 2 µM C_4_HSL. The plates were incubated at 37°C for 16 h to observe swarming motility [50].

#### Antagonistic activity

*E. coli* DH5α, harboring pSC11 (P*lasI*::*lacZ*) was electroporated with pJN105L (containing *lasR* gene) and used as bioreporter to check antagonistic activity of bioactive fraction. Overnight grown culture was diluted 1∶10 with fresh LB, incubated at 37°C and 150 rpm till OD_600 nm_ reached 0.3 [19]. Expression of *las R* was induced by addition of 4 mg/ml of arabinose. 10 µM 3-oxo-C_12_HSL was added along with a concentration gradient of bioactive fraction (0.2–1.0 mg/ml) and incubated further under similar conditions. β-Galactosidase activity was measured in the bioreporter strain as described [44].

### Purification and Characterization of Putative Active Compound(s)

50 µl of bioactive fraction was loaded on forward Silica Gel60F_254_ and separated into distinct spots using ethyl acetate, methanol and water (8∶1∶1) solvent system. The individual spots were scraped out and compounds were re-extracted from bound silica with a mixture of ethyl acetate: methanol (9∶1). The solvent was evaporated and residue was re-suspended in minimal volume of methanol. Each spot was then observed for QS inhibition using CVO26 [53].

The spot with anti QS activity was further analyzed using by LC ESI MS using Agilent 1100HPLC (HP 1101; Agilent technologies, Waldbronn, Germany). 20 µl of sample in methanol was injected into reverse phase C_18_ column (250×4 mm with i.d. 5 µm). The mobile phase consisted of 2% acetic acid in water (solvent A) and acetonitrile (solvent B) with following gradient profile: initially 95% A for 10 min; to 90% A for 1 min; to 80% A for 10 min; to 60% A for 10 min to 0% A for 5 min and continuing at 0% A until completion of the run. Mass spectra was obtained in the negative ion mode using 100 V fragmenter voltage and mass range of 100–1500D, Drying gas temperature was 350°C, capillary voltage 2500 Vand nebulizer pressure was 30 psi. Compounds were identified by comparing the standard masses and fragmentation peaks obtained with those available in literature for fruit of *Terminalia* species [35].

### Statistical Analysis

All the statistical analyses were performed using student t test and p<0.05 was considered significant.

## Results

### Anti QS Activity of *T. chebula* Extract

In disc diffusion assay, both aqueous (5 mg/ml) and methanol (1 mg/ml) extracts exhibited anti QS activity as shown by viable white colonies of A136 around the disc in a background of blue colonies (Fig. S1). As methanol extract showed anti QS activity at lower concentration, further experiments were done with it. Methanol extract did not bind or brought any structural change in AHL molecules as there was no significant difference (p = 0.125) in β-galactosidase activity of biosensor A136 when incubated with the extract-treated and untreated AHLs.

### Bioassay Guided Fractionation of *T. chebula* Methanol Extract

**Figure 1:**
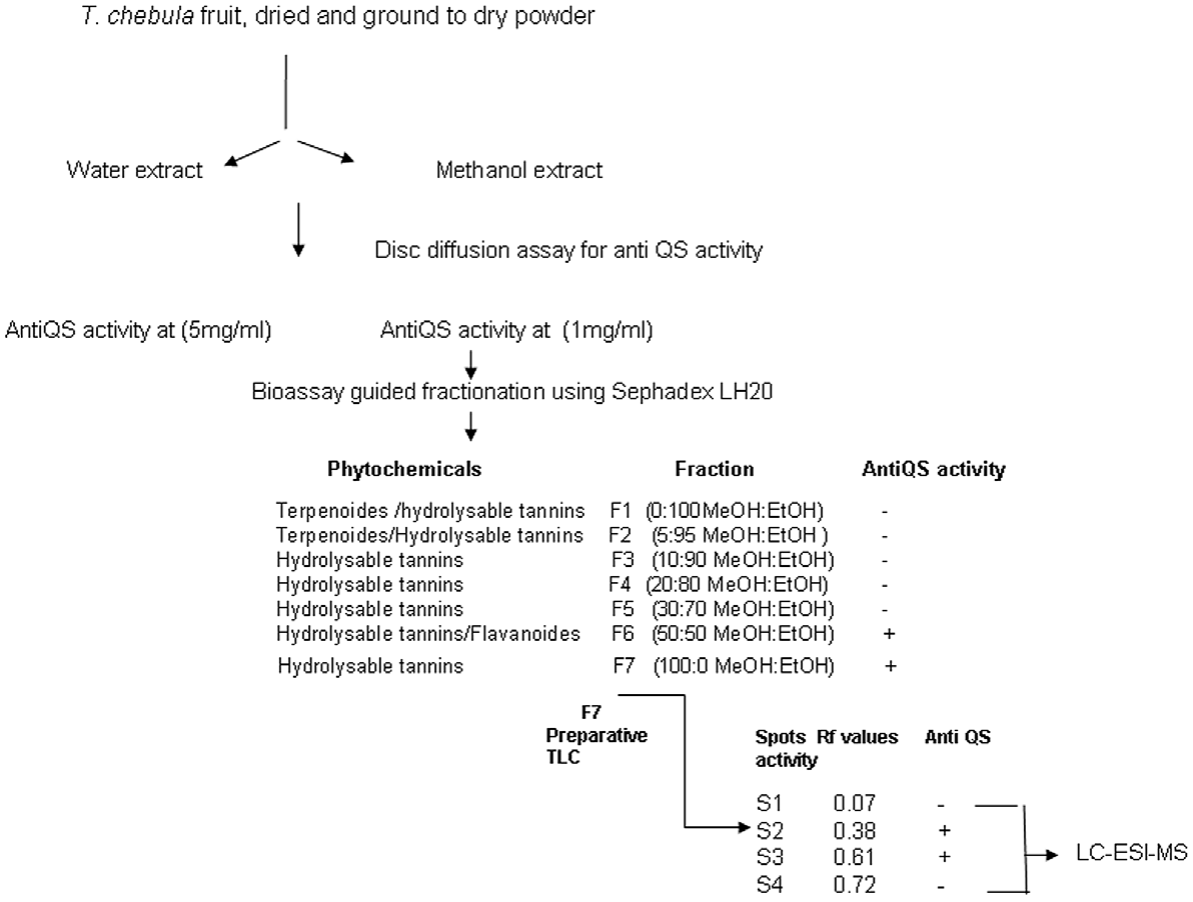
Schematic representation of bioassay guided fractionation of *T. chebula* fruit extract.

In order to separate the anti QS component(s), methanol extract fractionation scheme was followed as illustrated in Fig. 1. Fractions F6 and F7 showed anti QS activity indicated by 66 and 83% (p<0.001) reduction in violacein pigment production by biosensor CVO26, respectively (Fig. S2). However, anti QS components were probably more concentrated in F7 as it showed anti QS activity even at 0.1 mg/ml (data not shown). Phytochemical analysis of different fractions revealed the presence of terpenoides in F1 and F2, flavonoids in F6 and hydrolysable tannins in all seven fractions. However, protein precipitation assay confirmed the presence of higher amount of hydrolysable tannins in F4–F7 (Data not shown).

### Effect of F7 on the Production of Virulence Factors in *P. aeruginosa* PAO1

**Figure 2:**
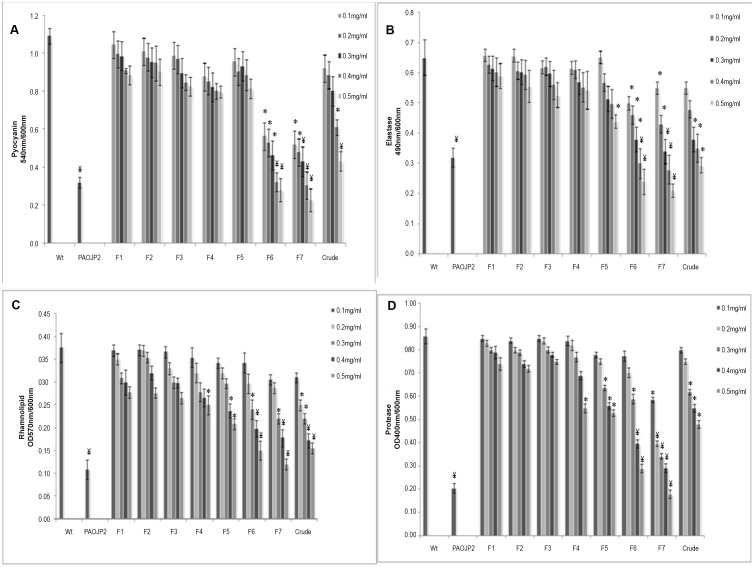
Effect of different fractions (F1–F7) on the production of virulence factors by *P. aeruginosa* PAO1 A)Pyocyanin B)Elastase C)Rhamnolipids D) Protease (* p<0.05, ¥ p<0.001). Bars indicate standard deviations for triplicate sets of experiments.

Significant reduction (p<0.001) in pyocyanin (60%), elastase (50%), rhamnolipids (58%) and protease (55%) production in the presence of 0.3 mg/ml of F7 was observed while F6 showed reduction (p<0.001) at 0.4 mg/ml. F4 and F5 did not affect the production of pyocyanin and elastase. However, there was reduction (p<0.05) in case of rhamnolipids and protease whereas F1-3 did not show any effect (Fig. 2). The virulence factors were reduced by the bioactive fraction to the level equivalent to that in PAOJP2. Alginate, an important component of extracellular polysaccharides of *P. aeruginosa* biofilm matrix and known to be regulated by QS was also significantly (p<0.05) reduced by 50% at 5 mg/ml (Fig. S3).

### Effect of F7 on Biofilm Formation by *P. aeruginosa* PAO1

**Figure 3:**
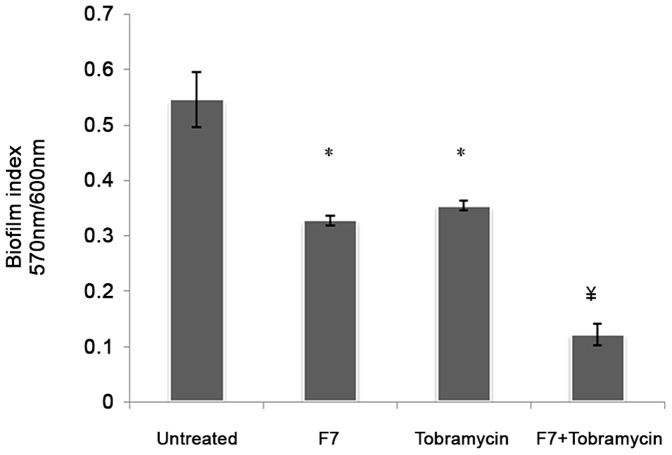
Effect of F7 (1 mg/ml) and tobramycin (20 µg/ml) on biofilm formation by *P. aeruginosa* PAO1.

**Figure 4:**
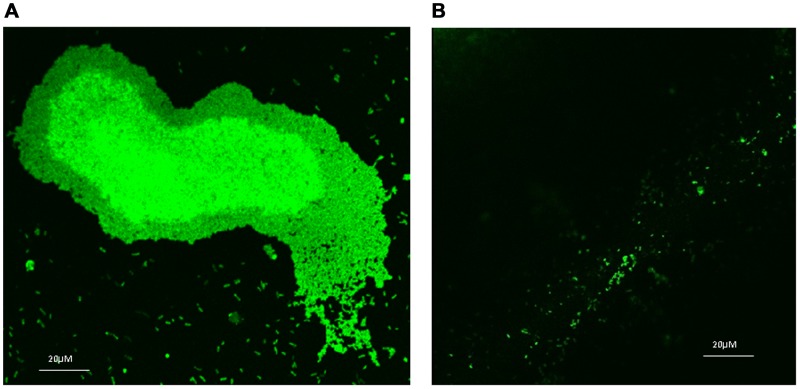
CLSM images of biofilm formed by *P. aeruginosa* PAO1 (63X magnification) A)Untreated B) Treated with 1 mg/ml F7.

Prophylactic efficiency of F7 was shown by significant reduction (65%, p<0.05) in biofilm formation in 24 h at 1 mg/ml (Fig. 3) that was increased to 85% at 5 mg/ml (p<0.001) (data not shown) with concomitant increase in planktonic bacterial cells by 1.2 log folds (from 2.75±0.78×10^6^ to 6.7±0.34×10^7^ CFU/ml). CLSM images of biofilm formed by GFP tagged *P. aeruginosa* in the presence of 1 mg/ml of F7 and stained with PI showed bacterial cells scattered singly on the adherent surface. Z stack analysis of the images showed 1–1.5 µm thickness that corresponds to size of single bacterial cell. However, in untreated sample, bacterial aggregation and formation of bacterial microcolonies was observed that measured to 10 µm. Non significant signal of PI indicated the absence of antibacterial effect of F7 and inhibition of biofilm formation was thus due to its anti QS activity (Fig. 4).

Minimum biofilm eradication concentration (MBEC) of tobramycin was found to be 100 µg/ml for *P. aeruginosa* PAO1. Tobramycin at sub inhibitory 20 µg/ml reduced biofilm formation by 60% (p<0.05) after 24 h. However, when tobramycin (20 µg/ml) was used along with F7 (1 mg/ml) biofilm was reduced by 80% (p<0.001) showing enhanced susceptibility to tobramycin (Fig. 3).

### *C. elegans* Killing Assay

**Figure 5:**
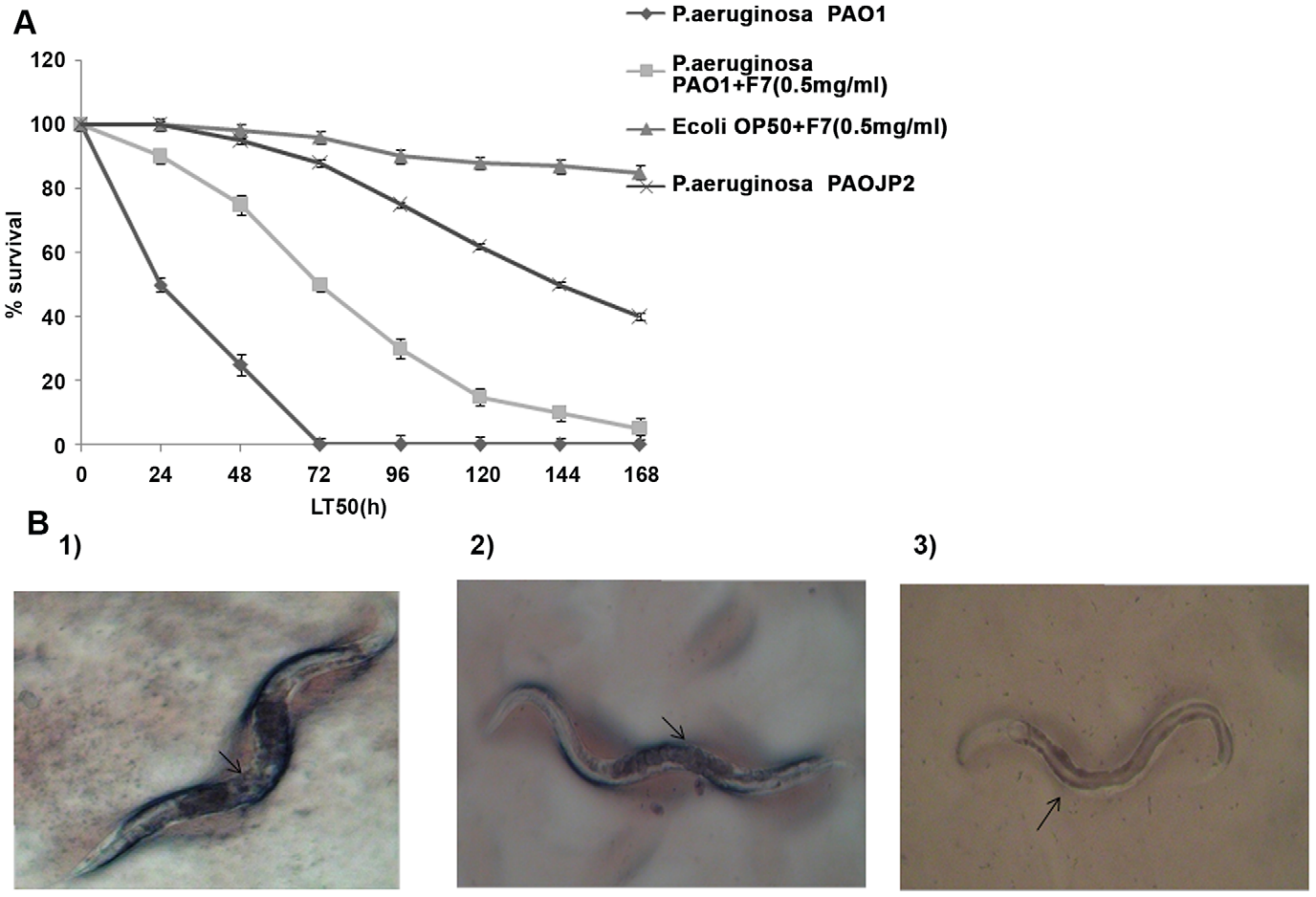
*C. elegans*- *P. aeruginosa* killing assay A) LT _50_ of *C. elegans* increased from 24 to 72 h when fed on *P. aeruginosa* PAO1 treated with 0.5 mg/ml of F7. B) Microscopic images of *C. elegans* (100X) fed on 1) *P. aeruginosa* PAO1 2) *P. aeruginosa +*0.5 mg/ml F7 3) *E. coli* OP50*+*0.5 mg/ml F7.

*C. elegans* N2 fed on *P. aeruginosa* PAO1 showed decrease in motility within 12 h of incubation that subsequently led to death of 50% worm (LT50) within 24 h. However, LT50 increased to 72 h when worms were allowed to feed on F7 (0.5 mg/ml) treated *P. aeruginosa* PAO1 and an increase was also seen with PAOJP2 an autoinducer deficient mutant (LT_50_ 144 h) (Fig. 5A). Microscopic examination of *P. aeruginosa* fed *C. elegans* showed distention of gut as possible indication of infection like process. This distention was also present in worms fed on F7 treated *P. aeruginosa* but exhibited prolonged survival rates (Fig. 5B1and 5B2). Analysis of bacterial load in the worm gut after 24 h of feeding on *P. aeruginosa* PAO1 did not show significant difference (p = 0.198) (5±0.31×10^6^ CFU/ml/worm in untreated and 8±0.58×10^6^ CFU/ml/worm in treated groups) indicating the attenuation of virulence of *P. aeruginosa* PAO1 colonizing worm gut, without affecting its viability. *C. elegans* fed on *E. coli* OP50 in the presence of F7 (0.5 mg/ml) had normal physiology with proper egg laying,life cycle (2.5 days at 25°C), motility and intact gut morphology (Fig. 5B3) showing absence of F7 toxicity.

### Mechanism of Anti QS Activity of F7

**Figure 6:**
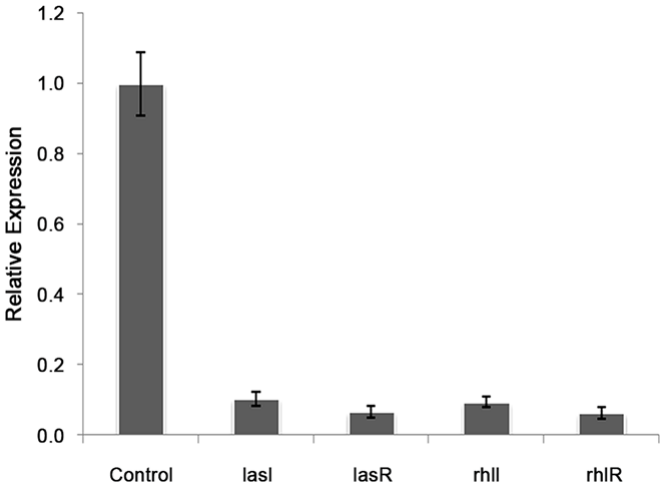
Relative expression of *lasIR* and *rhlIR* genes of *P. aeruginosa* PAO1 in the presence of 0.5 mg/ml F7 as determined by qRT PCR.

**Figure 7:**
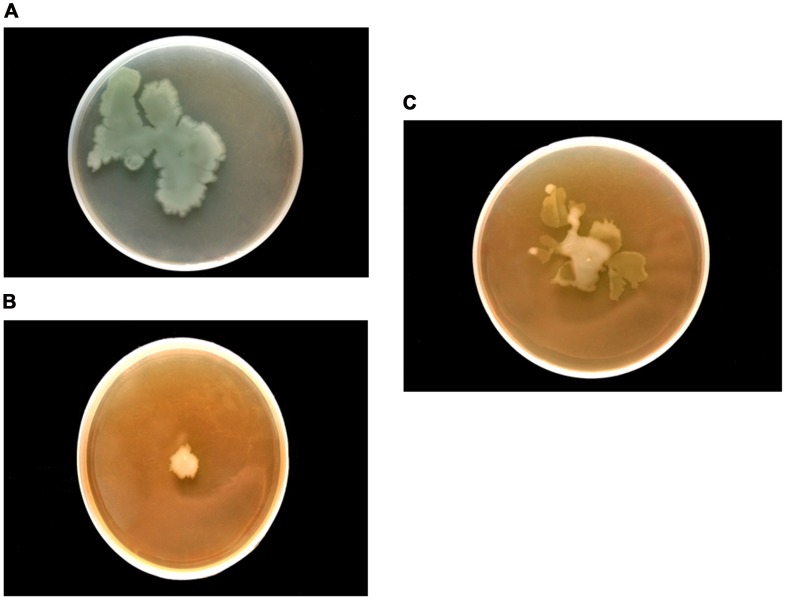
Swarming motility of *P. aeruginosa* PAO1 a) Untreated b) Treated with 0.5 mg/ml of F7 c) reversal of inhibited swarming motility by the addition of exogenous C_4_HSL(2 µM).

**Figure 8:**
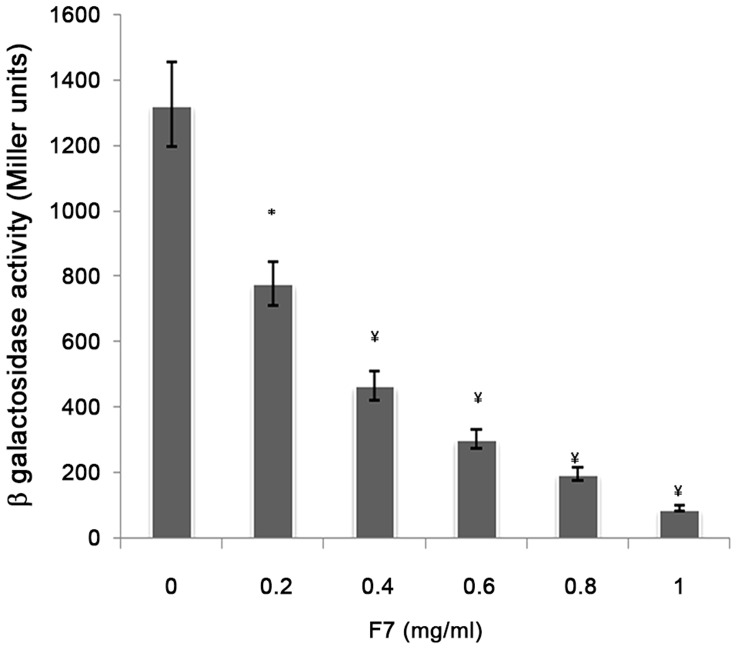
Antagonistic activity of F7 against 3-oxo-C_12_HSL mediated QS in *E. coli* bioreporter strain harboring pSC11(P*lasI*)*::lacZ*) and lasR expression vector pJN105L.

Real time PCR showed 89, 90, 90 and 93% reduction in the expression of *lasI*, *lasR*, *rhlI* and *rhlR,* respectively with 0.5 mg/ml of F7(Fig. 6). This was supported by the reduction of peak intensity of 3-oxo-C_12_HSL by 64% (peaks corresponding to 3-oxo-C_12_HSL at m/z 316 ammonium and 595 dimer adduct) and 90% reduction in C_4_-HSL (peaks corresponding to C_4_HSL at m/z 159) on ESI MS analysis of AHLs after F7 exposure (Fig. S4). The reduction in both AHLs was consistence with the reduction in virulence factors controlled by 3-oxo-C_12_HSL (elastase and protease) and C_4_HSL (rhamnolipids and pyocyanin) in *P. aeruginosa* PAO1(Fig. 2). Growth of *P. aeruginosa* PAO1 was monitored in the presence of different concentrations of F7 (0–1.25 mg/ml) and it showed insignificant change in growth (p = 0.132) at 0.5 mg/ml (Fig. S5) indicating quorum sensing inhibition as the mechanism for the reduction in AHLs and not the killing of cells. Restoration of inhibited swarming motility on addition of 2 µM C_4_HSL further supported the observation (Fig. 7). F7 at 1 mg/ml also reduced β-galactosidase activity by 93% in *E. coli* bioreporter strain showing its antagonistic activity towards transcriptional regulator lasR (Fig. 8).

### Identification of Bioactive Constituents

TLC of F7 using silica gel 60 F_254_ resolved into four different spots (S1, S2, S3 and S4) with Rf value of 0.07, 0.38, 0.61 and 0.72. Anti QS activity was found in S2 and S3 using CVO26 (data not shown). LC-ESI-MS analysis of S2 showed three peaks at RT of 1.26, 13.36, 34.63 min. On comparing the peaks with the reported MS fragmentation data of polyphenols from *Terminalia* species (Table S1) [35], peak obtained at RT 1.26 min may be of 3-O-methyl-4-O-(β–D-xylopyranosyl) ellagic acid, that is a glycosylated derivative of ellagic acid with m/z peaks of 447,315,126,217 representing C_20_H_16_O_12_ (mol wt. 448)_._ RT 13.36 min peak probably corresponded to ellagic acid molecule with molecular formula of C_14_H_6_O_2_ (mol wt. 302) and [m/z+H^−^] of 283,255,243,200,173 whereas peak at RT 34.63 min could be the methylated derivative of (S)-flavogallonic acid C_22_H_12_O_13_ (mol wt.485) with [m/z+H^−^] of 323, 255,227,200 (Table 2). In S3 fraction, three RT peaks were identified at 0.74,13.63, 34.63 min. Peak at RT 0.74 min may be of (S)-flavogallonic acid C_21_H_10_O_13_ (mol wt.470) that gave [m/z+H^−^] peak of 469. (S)-flavogallonic acid possesses both ellagic acid and gallic acid moieties. Therefore, [m/z+H^−^] peaks of both gallic acid at 126 and ellagic acid at [m/z+H^−^] 305, 217,145 were observed. Peak at RT 13.63 min corresponded to the molecular formula of C_20_H_16_O_12_ (mol wt. 276) that is 3,4,8,9,10–pentahydroxyldibenzo(b,d)pyran-6-one molecule and showed m/z of 276,255,227,201,173 that corresponded to the standard MS-MS fragmentation data of this molecule. Peaks at RT 34.63 min showed [m/z+H^−^] of 393, 293, 265, 106. The nucleus of this compound consists of C_3_H_6_0_3_ as the major [m/z+H^−^] peak observed was at 106. However, exact chemical formula could not be elucidated.

**Table 2:** Putative anti QS compounds as shown by LC-ESI-MS fragmentation data for the bioactive fraction.

Proposed compounds	MolecularFormula	MolecularWeight	Retention Time(min)	(M+H)^−^	MS-MS fragmentationPeaks
**Spot S2**					
**3-O-methyl-4-O-(β-D-xylo pyranosyl)ellagic acid**	C_20_H_16_O_12_	448	1.26	447	447,315, 217,126
**Ellagic acid**	C_14_H_6_O_2_	302	13.36	301	255,283,243,200,173
**Methyl S-flavogallonic acid**	C_22_H_12_O_13_	485	34.63	484	323,255,227,200
**Spot S3:**					
**S flavogallonic acid**	C_21_H_10_O_13_	470	0.74	469	305,217, 145,126
**3,4,8,9,10-pentahydroxyldibenzo(b.d) Pyran-6-one**	C_20_H_16_O_12_	276	13.63	275	276,255,227,201,173
**Unknown**	Unknown	Unknown	34.63	Unknown	106,265,293,393

## Discussion

Plant products have been used traditionally for the treatment of various ailments and this is attributed to the wide range of phytochemicals present in them. In the present study, both aqueous and methanol extract showed anti QS activity. However, methanol extract exhibited greater anti QS activity indicating that effective phytochemical compound(s) have been partitioned more in organic phase. In most of the studies, organic extracts of plants have showed anti QS activity as reported in toluene extracts of *Allium sativum* [54], ethanol extracts of *C. arbiflorum* leaves [1], *Mangifera indica* and *Puncia granatum* [55].

Methanol extract of *T. chebula* on partial purification led to the separation of hydrolysable tannins as a group of phytochemicals responsible for the anti QS activity. Tannins in plants protect them from predators and also play role in plant growth regulation [56]. Initially tannins were regarded as anti nutritional but due to their antioxidant and antimicrobial properties, they are now being used in various medicinal formulations [57]. Ellagic acid, gallic acid, corilagen, chebulagic acid and punicalagin are some of the known polyphenolics isolated from *T. chebula* fruit [21]. Anti QS activity has been linked only with ellagic acid which reduced swarming motility and biofilm formation in soil isolate, *P. putida* [16]. However, ellagic acid has not been shown to attenuate *P. aeruginosa* virulence. In the present study, LC-ESI-MS analysis of tannin-rich bioactive fraction showed the presence of ellagic acid derivatives (EADs) as major compounds. EADs constitute the polyphenolic compounds that possess ellagic acid as the core molecule. Bioactive fraction, on mass identification, revealed the presence of glycosides derivatives of ellagic acid. EADs extracted from *Rubus ulmifolius* along with some sapogenin related compounds have been shown to reduce biofilm formation in *Staphylococcus aureus* with enhancement to antibiotics (Clindamycin, Daptomycin, Oxacillin) susceptibility [58]. To the best of our knowledge, anti QS activity of ellagic acid derivatives from *T. chebula* and its use to control *P. aeruginosa* virulence has not been studied before.

Pyocyanin, elastase, protease and rhamnolipids are regarded as indicators of the optimal operation of QS regulon in *P. aeruginosa*. Reduction in their production level indicates the anti QS potential of the tested compound(s). Elastase and protease form important determinants in colonizing the host tissues [59] whereas pyocyanin chelates the bound iron from transferrin for optimal virulence expression [60]. Rhamnolipids constitute an important surfactant that assists in surface motility of *P. aeruginosa* required for biofilm initiation [61]. Bioactive fraction was able to reduce all of them to the level comparable in *lasI* ^−^ *rhlI* ^−^ mutant PAOJP2, indicating the effectiveness of EADs in attenuating *P. aeruginosa* virulence factors.

Alginate is one of the important virulence determinants in *P. aeruginosa* and is present as a constituent of exopolysacchrides (EPS) in biofilms. [62]. Tannic acids have been reported to cause massive decrease in EPS production in *Streptococcus* species [63] which may explain the decrease in alginate content in *P. aeruginosa* biofilm after treatment with bioactive fraction that contains hydrolysable tannins. This also increased the planktonic bacterial cell count in the medium surrounding biofilm making them vulnerable to the action of tobramycin.

*In vitro* attenuation of virulence factors correlated well with the *in vivo* study. Slow killing of *C. elegans* occurs due to colonization and proliferation of bacteria in the worm gut [64]. CFU analysis of worm gut fed on treated and untreated *P. aeruginosa* showed that bioactive fraction was able to attenuate the virulence of colonizing bacteria indicating therapeutic potential of the fraction. Phenazines, produced by *P. aeruginosa* causes lethal paralysis of muscular tissues in *C. elegans* leading to asphyxia and death of worms within 4–24 h. F7 was found to inhibit the pyocyanin (phenazine) pigment production that may be the reason for increase in LT50 of worms fed on treated *P. aeruginosa.*

QS can be inhibited in various ways. Halogenated furanones or synthetic analogs act as signal mimics resulting in a decrease in QS gene expression [20]. Lactonases and acylases from Gram positive bacteria cause enzymatic degradation of AHLs resulting in inhibition of QS in Gram negative bacteria [65]. QscR a negative transcriptional regulator homolog of *lasR* and *rhlR* in *P. aeruginosa* also led to similar inhibition of QS [41]. Lactonolysis, a pH meditated degradation of AHLs inhibits QS [66]. As pH of *T. chebula* extract was neutral hence there was no spontaneous inactivation of AHLs. EADs-rich bioactive fraction inhibited QS by reducing AHLs production as shown in the present study. In the previous studies, anti biofilm activity of tannin rich fraction from *T. cattapa* has been reported but the underlying mechanism has not been explained [34]. Similarly, proanthocyanidins (condensed tannins from cranberries) has been reported to inhibit swarming motility in *P. aeruginosa* by multiple mechanisms that include binding with lipopolysacchrides, flagellin subunits, and reduction in rhamnolipids production [67] but their effect on *lasIR* and *rhlIR* remained unexplored. Plants extracts of *Conocarpus erectus*, *Callistemon viminalis*, *Bucida buceras* and *Combretum arbiflorum* have been reported to downregulate the expression of *lasIR* and *rhlIR* using bioreporter strains whereas in the current study, qRT PCR analysis provides more specific analysis of QS gene expression in *P. aeruginosa* treated with bioactive fraction of *T. chebula*. Antagonist activity of synthetic QS inhibitor *viz*. isothiocyanate and haloacetamides has been reported as these compounds displace 3-oxo-C_12_HSL from the ligand binding domain of lasR [19]. Antagonist activity was also displayed by the bioactive fraction of *T. chebula.*

**Figure 9:**
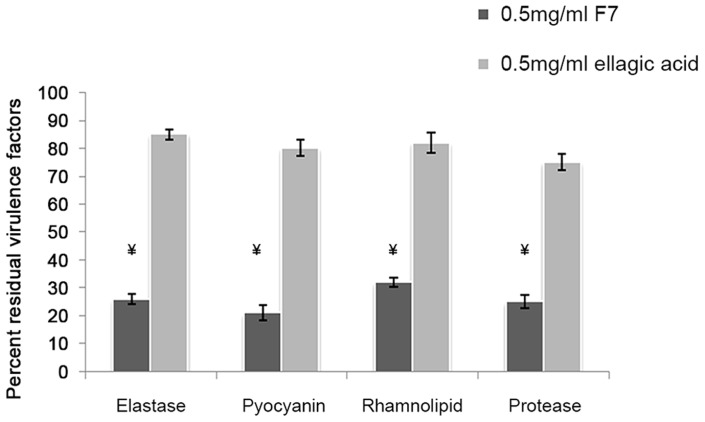
Comparative effect of F7 and ellagic acid on the production of virulence factors at 0.5 mg/ml. Elastase OD_490 nm/600 nm_ 0.654,Pyocyanin OD _540 nm/600 nm_ 1.08, Rhamnolipids OD _570 nm/600 nm_ 0.456 and Protease OD _400 nm/600 nm_0.876 were taken as 100% in untreated *P. aeruginosa* PAO1.

Since ellagic acid formed the core molecule in F7, effect of ellagic acid was checked on *P. aeruginosa* virulence factors. There was only 10–15% reduction in virulence factors production at concentration of 0.5 mg/ml (Fig. 9). In contrast, F7 was able to show more than 50% reduction in virulence factors production in *P. aeruginosa* PAO1 at the same concentration suggesting the importance of ellagic acid derivatives in the inhibition of QS in *P. aeruginosa* PAO1. Further studies are required to find out which EADs have antagonistic activity that act by down regulating the *lasIR* and *rhlIR* system. The combination of EADs needs to be worked out to develop an effective anti QS formulation to control AHL mediated virulence in pathogens.

## Supporting Information

Figure S1 **Disc diffusion assay for anti quorum sensing activity of** ***T. chebula*** **using** ***A. tumefaciens*** **A136 as biosensor.** 1. Aqueous extract (5 mg/ml) 2. Methanol extract (1 mg/ml) 3.Curcumin (3 µg/ml ) as positive control 4.Methanol and 5.Water as negative controls.
(TIF)

Figure S2 Anti quorum sensing activity of different fractions at 0.5 mg/ml shown as reduction in violacein production by CV026 in the presence of 50 nM C_6_HSL (*∼p<0.05, ¥∼p<0.001 ). Bars indicates standard deviations for triplicate sets of experiments.
(TIF)

Figure S3 **Effect of F7(1–10 mg/ml) on alginate content in biofilms of** ***P. aeruginosa*** **PAO1.**
(TIF)

Figure S4 ESI-MS analysis of AHLs extracted from *P. aeruginosa* PAO1 A) untreated B) treated with 0.5 mg/ml of F7.
(TIF)

Figure S5 **Effect of F7 (0–1.25 mg/ml) on growth of** ***P. aeruginosa*** **PAO1.**
(TIF)

Table S1 Compound(s) isolated from *T. chebula* fruit [35].
(TIF)
