# Supplementary figures and images for: Ellagic Acid Derivatives from Terminalia chebula Retz. Downregulate the Expression of Quorum Sensing Genes to Attenuate Pseudomonas aeruginosa PAO1 Virulence
Source: PLoS One. 2013 Jan 8;8(1):e53441. doi: 10.1371/journal.pone.0053441 (PMC3539995; doi:10.1371/journal.pone.0053441)

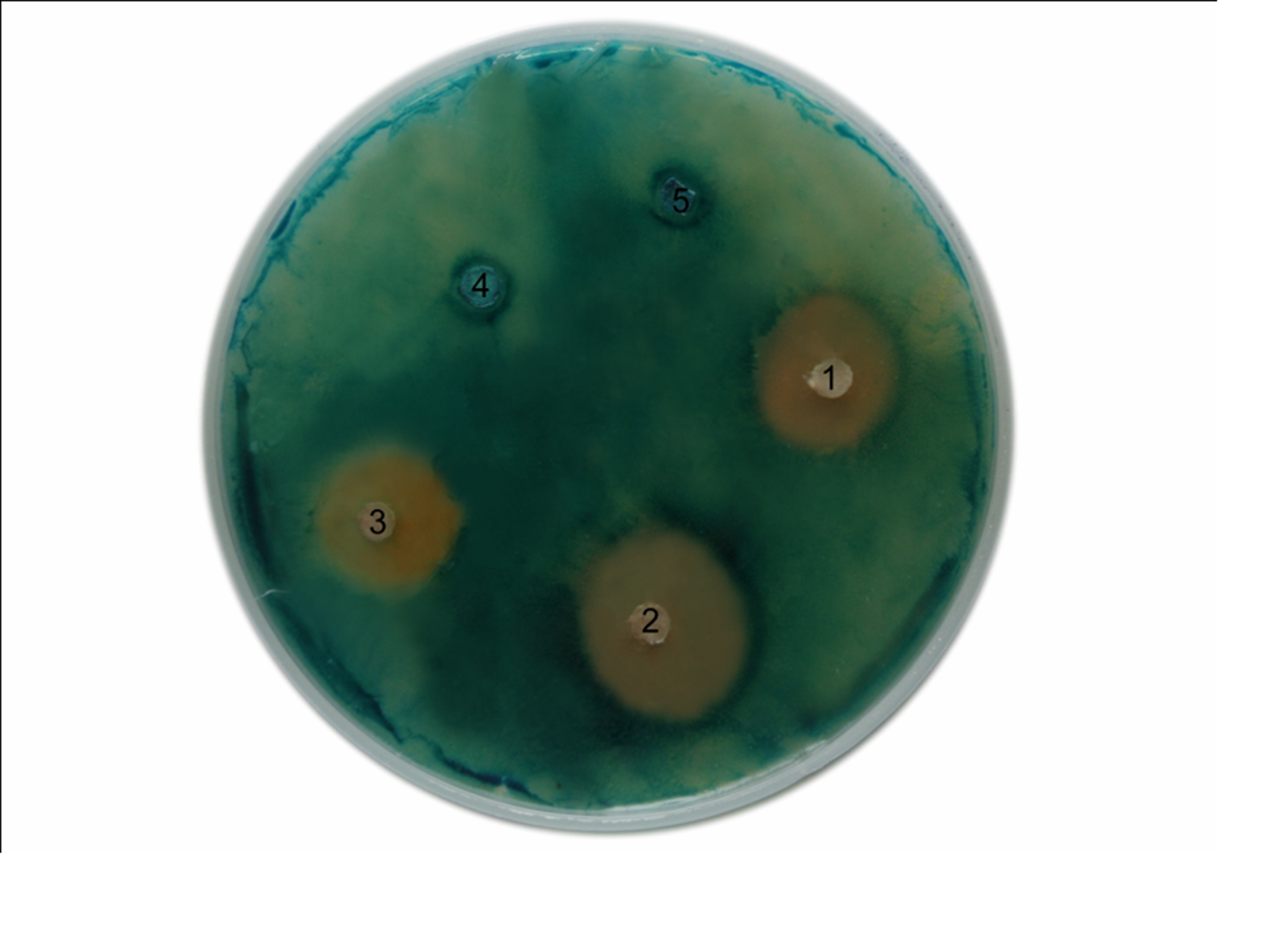

Supplement: Figure S1 — Disc diffusion assay for anti quorum sensing activity of T. chebula using A. tumefaciens A136 as biosensor. 1. Aqueous extract (5 mg/ml) 2. Methanol extract (1 mg/ml) 3.Curcumin (3 µg/ml ) as positive control 4.Methanol and 5.Water as negative controls. (TIF) [file pone.0053441.s001.tif]

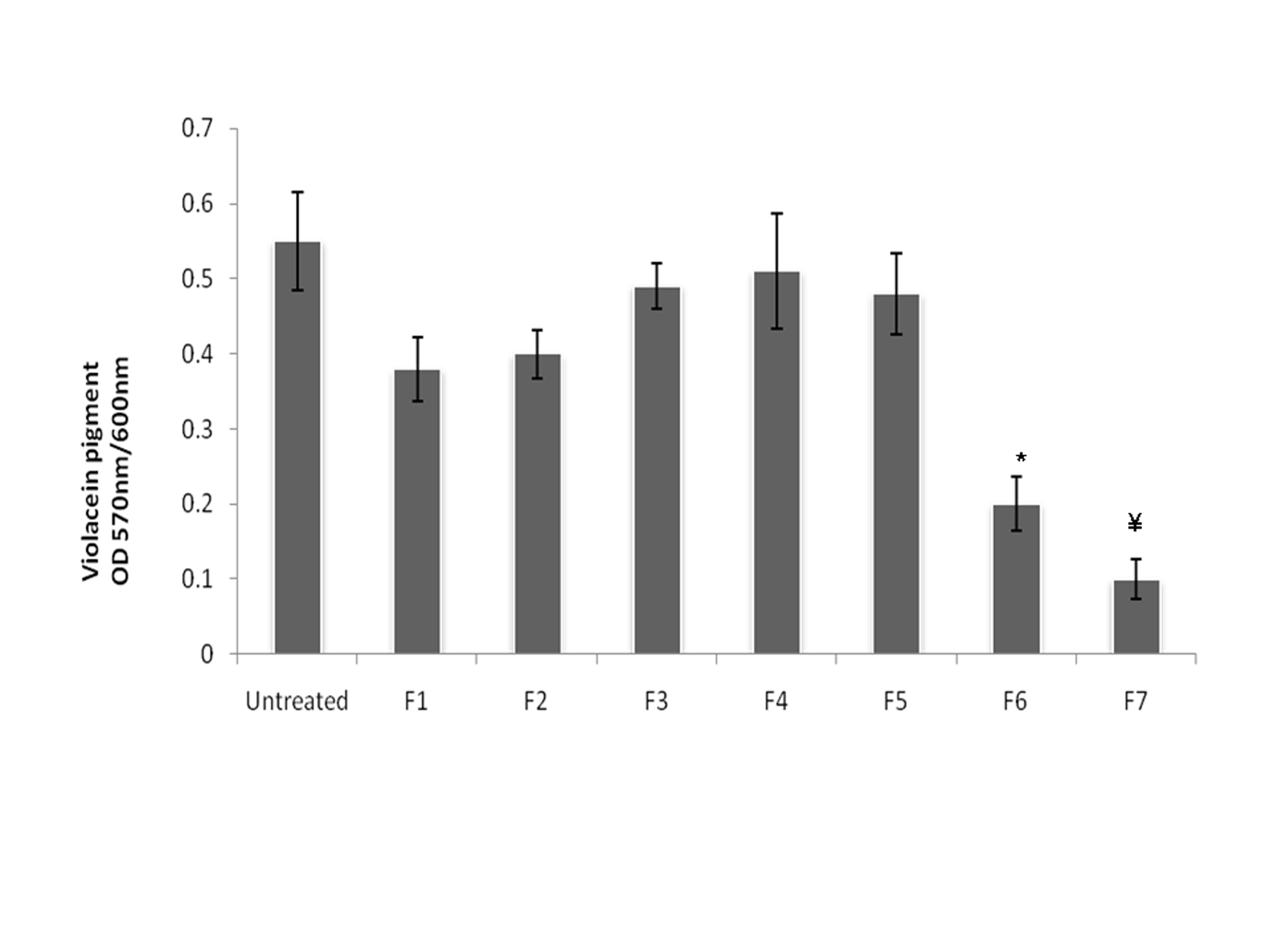

Supplement: Figure S2 — Anti quorum sensing activity of different fractions at 0.5 mg/ml shown as reduction in violacein production by CV026 in the presence of 50 nM C6HSL (*∼p<0.05, ¥∼p<0.001 ). Bars indicates standard deviations for triplicate sets of experiments. (TIF) [file pone.0053441.s002.tif]

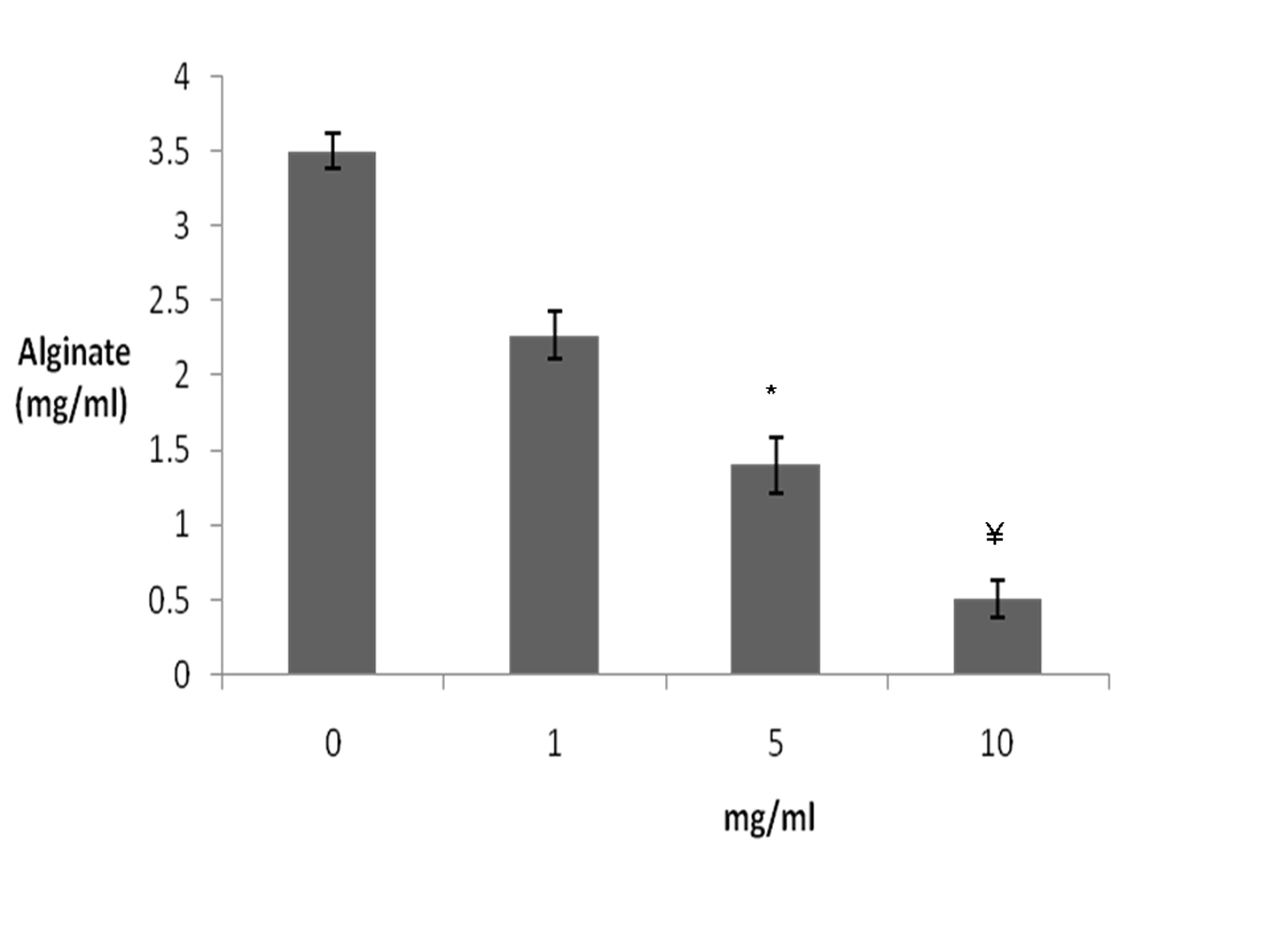

Supplement: Figure S3 — Effect of F7(1–10 mg/ml) on alginate content in biofilms of P. aeruginosa PAO1. (TIF) [file pone.0053441.s003.tif]

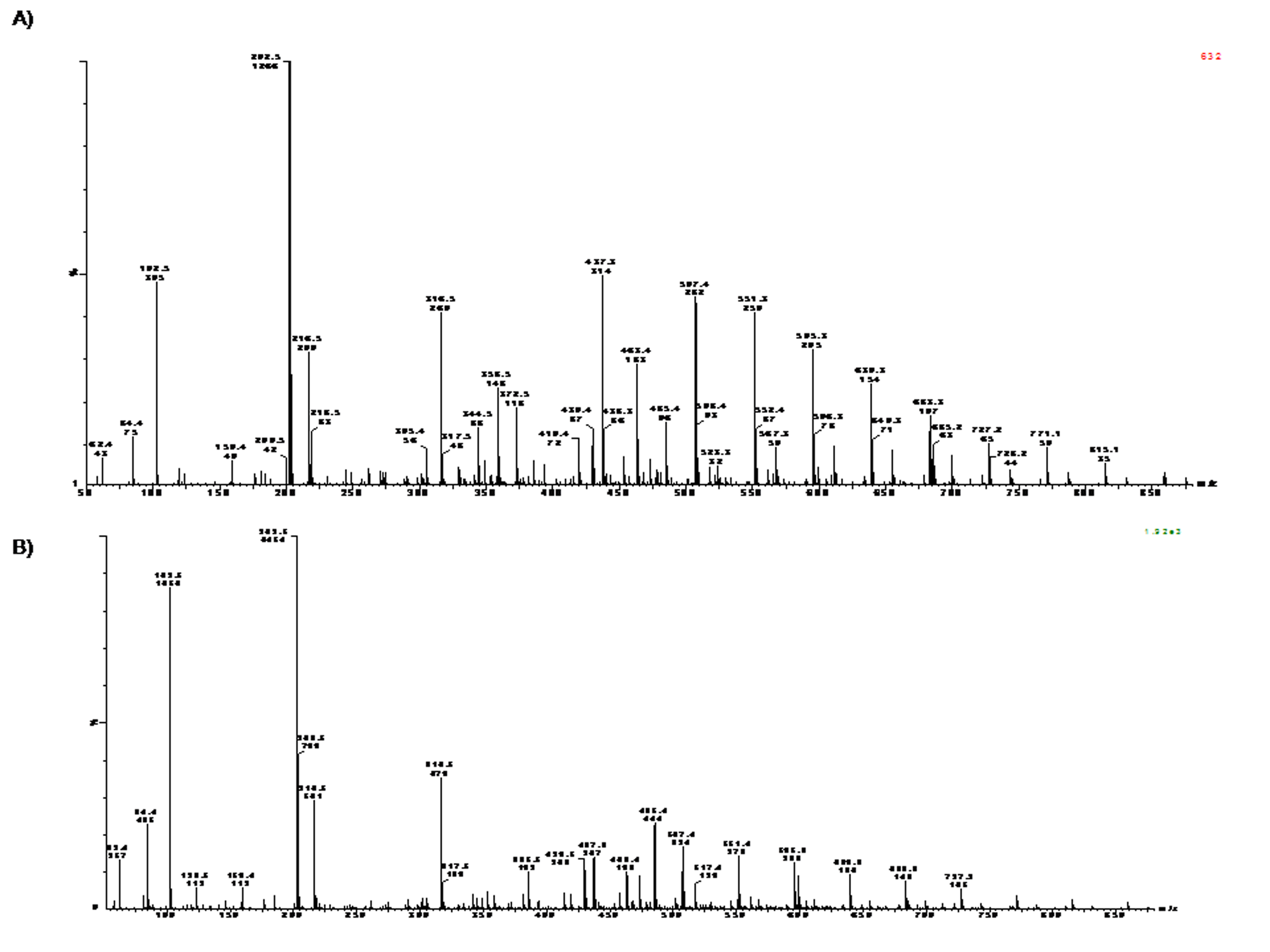

Supplement: Figure S4 — ESI-MS analysis of AHLs extracted from P. aeruginosa PAO1 A) untreated B) treated with 0.5 mg/ml of F7. (TIF) [file pone.0053441.s004.tif]

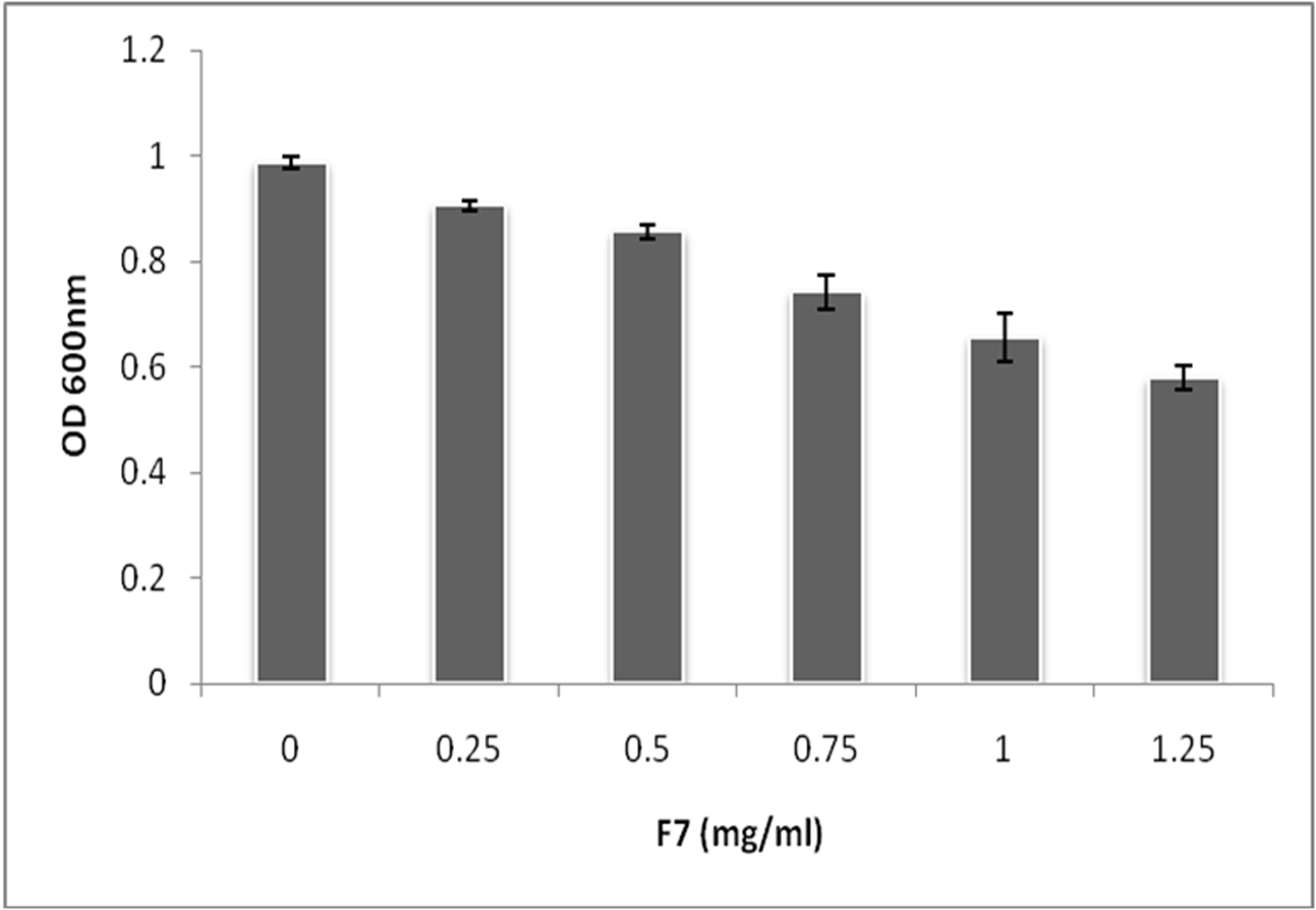

Supplement: Figure S5 — Effect of F7 (0–1.25 mg/ml) on growth of P. aeruginosa PAO1. (TIF) [file pone.0053441.s005.tif]

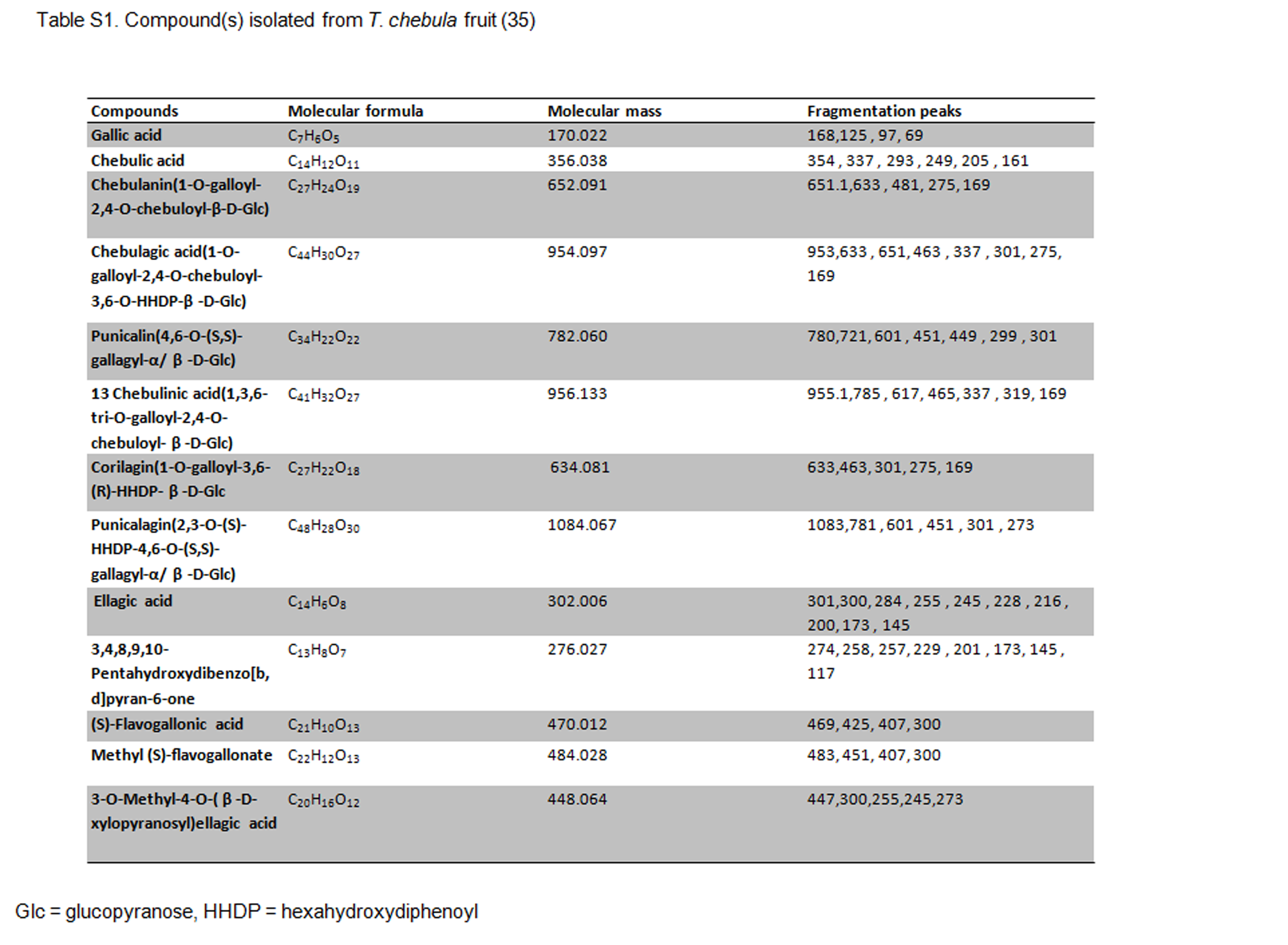

Supplement: Table S1 — Compound(s) isolated from T. chebula fruit [35]. (TIF) [file pone.0053441.s006.tif]
